# Erratum

**Published:** 2015-03-13

## Vol. 62, No. RR-4

In the *MMWR* Recommendations and Reports, “Prevention of Measles, Rubella, Congenital Rubella Syndrome, and Mumps, 2013: Summary of Recommendations of the Advisory Committee on Immunization Practices (ACIP),” an error occurred in the third sentence of the first paragraph under the heading “Measles Component” on page 8. That sentence should read:

“Because of increased efficacy and fewer adverse reactions, the vaccine containing the Enders-Edmonston vaccine strain replaced previous vaccines: inactivated Edmonston vaccine (available in the United States from 1963 through **1967**), live attenuated vaccines containing the Edmonston B (available in the United States from 1963 through 1975), and Schwarz strain (available in the United States from 1965 through 1976).

